# Transfer of 4-hydroxynonenal, a inhibitory hemozoin (HZ) product, from HZ or HZ-laden phagocytes to developing human erythroid cells. A model for erythropoiesis inhibition in malaria anemia

**DOI:** 10.1186/1475-2875-9-S2-O28

**Published:** 2010-10-20

**Authors:** Oleksii A Skorokhod, Luisa Caione, Giorgia Migliardi, Valentina Barrera, Wanda Piacibello, Evelin Schwarzer, Paolo Arese

**Affiliations:** 1 Department of Genetics, Biology and Biochemistry, University of Torino Medical School, Torino 10126, Italy; 2 Institute for Cancer Research and Treatment, Laboratory of Cell Therapy, University of Torino Medical School, Torino 10126, Italy

## 

Blood stage *P. falciparum* (Pf) accumulates hemozoin (HZ), the crystal of dimerized undigested heme. HZ wrapped up in the food vacuole membrane binds saturated and polyunsaturated fatty acids (PUFA) and proteins, generates bioactive ligands and is expelled in the blood during schizogony as residual body. HZ contains redox active iron that peroxidizes PUFA to produce bioactive hydroxy-PUFA (HETEs; HODEs) and terminal aldehydes (4-hydroxynonenal, HNE). HETEs is responsible for some of the HZ effects: (1) down-modulation of oxidative burst; (2) inhibition of dendritic cells (DC) differentiation-maturation; and (3) enhancement of expression and enzyme activity of metalloproteinase MMP-9. Low-micromolar HETE recapitulated effects (2) and (3). Recapitulation was abrogated by PPAR-gamma-receptor inhibitors.

Other effects, such as inhibition of erythropoiesis, were due to the transfer of HNE to differentiating human erythroid precursors. Erythropoiesis was inhibited by transferred HNE via blockage of cell cycle and the down-regulation of protein expression of crucial receptors (erythropoietinR, transferrinR and stem-cell-factorR). Malarial dyserythropoiesis, which plays an important role in malaria anaemia (MA) is characterized by (1) decreased growth and differentiation of erythroid precursors; (2) low reticulocyte response; and (3) normal production of erythropoietin (EPO). In human bone marrow (BM) the erythroblastic island (EI) is the elementary unit of erythropoiesis in which a central macrophage (MAC) is surrounded by differentiating erythroid cells. In malaria anaemia, HZ-laden central MACs are closely adjacent to developing erythroid cells. HZ and HZ-laden human monocytes inhibited growth of co-cultivated human erythroid cells and produced HNE that diffused to adjacent cells generating HNE-protein adducts. Co-cultivation with HZ or treatment with low-micromolar HNE inhibited growth of erythroid cells interfering with cell cycle without apoptosis. Following HZ/HNE treatment, two critical proteins in cell cycle regulation, p53 and p21, were increased and the retinoblastoma protein, central regulator of G1-to-S-phase transition, was consequently hypophosphorylated, while GATA-1, master transcription factor in erythropoiesis was reduced. The resul was decreased expression of cyclin A and D2 retarded cell cycle progression in erythroid cells and the K562 cell line. As a second major effect, HZ and HNE inhibited protein expression of crucial receptors (R): transferrin R1, stem cell factorR, interleukin-3R and erythropoietinR. The reduced receptor expression and the impaired cell cycle activity decreased the production of cells expressing glycophorin-A and hemoglobin.

In conclusion, present data confirm the inhibitory role of HZ, identify HNE as one HZ-generated inhibitory molecule and describe molecular targets of HNE in erythroid progenitors possibly involved in erythropoiesis inhibition in malaria anemia.

Supported by Regione Piemonte, IARC and Compagnia di San Paolo grants to ES, WP and PA.

